# Endemicity and environmental reservoirs of schistosomiasis and soil-transmitted helminth infections in Ghanaian rural communities

**DOI:** 10.1371/journal.pntd.0014701

**Published:** 2026-09-03

**Authors:** Rita Nyaaba Akologo, Isaac Williams, Dennis Adu-Gyasi, Dodzi Kofi Amelor, Rabbi Coffie Baidoo, Asenso Gideon, Akugre Alfred Akuka, Akua Obeng Forson, Samuel Fosu Gyasi, Emmanuel Timmy Donkoh

**Affiliations:** 1 Centre for Research in Applied Biology, University of Energy and Natural Resources, Sunyani, Ghana; 2 Kintampo Health Research Centre, Research and Development Division, Ghana Health Service, Kintampo, Ghana; 3 National Public Health and Reference Laboratory, Ghana Health Service, Accra, Ghana; 4 Department of Medical Laboratory Science, University of Ghana, Accra, Ghana; 5 Department of Biological Science, University of Energy and Natural Resources, Sunyani, Ghana; 6 Department of Medical Laboratory Science, University of Energy and Natural Resources, Sunyani, Ghana; Universidad de la República Uruguay: Universidad de la Republica Uruguay, URUGUAY

## Abstract

**Background:**

Schistosomiasis and soil-transmitted helminth (STH) infections remain major neglected tropical diseases in low- and middle-income countries despite ongoing preventive chemotherapy programmes. This study assessed the prevalence, associated risk factors, and household environmental contamination related to schistosomiasis and STH infections among children in Banda District, Ghana.

**Methodology:**

A community-based cross-sectional study was conducted in four endemic communities in the Banda District, Ghana, from February to April 2025. Pre-school and school-aged children (2–15 years) were recruited using systematic sampling. Urine samples were examined with urine sedimentation for *S. haematobium ova,* and stool samples were analyzed using Kato-Katz for STHs and *S. mansoni ova*. Household soil and animal faecal samples were examined using flotation and formalin-ether concentration techniques. Risk factors were assessed through structured questionnaires. Bivariate and multivariate logistic regression analyses were performed; variables with p ≤ 0.25 were included in the multivariate model, with a p < 0.05 considered statistically significant.

**Results:**

The overall prevalence of schistosomiasis was 26.3%, and the prevalence of STH infections was 28.0%. In multivariate analysis, non-school attendance (AOR = 4.59; 95% CI: 2.49-8.47; p < 0.001) and children drinking from rivers/streams (AOR = 4.58; 95% CI: 1.73 – 12.10; p = 0.009) were associated with a higher odds of schistosomiasis. For STH infections, children aged 2–9 years (AOR = 2.18; 95% CI: 1.24–3.85; p = 0.007), non-school attendance (AOR = 5.91; 95% CI: 3.41–10.24; p < 0.001) and rearing of animal within house (AOR = 4.78; 95% CI: 2.32–9.86; p < 0.001) were associated with a higher odds of infection. Both soil and animal faecal samples harbored STHs with differing species distributions between matrices (soil: 7.08% positive; animal faeces: 10.67%).

**Conclusion:**

This study revealed that schistosomiasis and helminth infections are prevalent among humans, animals, and the surrounding soil within shared environments. The findings support extending MDA to the community level and implement WASH, improved livestock management and health education to reduce transmission.

## Author summary

Schistosomiasis and intestinal parasite infections are among the major neglected tropical diseases affecting children in communities with limited access to clean water, sanitation, and hygiene. These infections, caused by parasites such as *Ascaris lumbricoides*, *Trichuris trichiura*, *Strongyloides stercoralis*, and hookworms and schistosome parasites, remain widespread in many low- and middle-income countries, particularly in sub-Saharan Africa. They contribute to poor child health, malnutrition, impaired growth, reduced school attendance, and poor academic performance. Although preventive chemotherapy programmes, including annual mass drug administration with praziquantel and albendazole, are ongoing in Ghana, transmission continues in many endemic areas. This study investigated schistosomiasis and intestinal worm infections among children living in selected endemic communities in the Banda District of Ghana. We found that 26.3% of children had schistosomiasis, while 28.0% were infected with at least one intestinal worm parasite. Children who did not attend school, practiced poor hygiene, and used contaminated river were more likely to be infected. We also detected parasite contamination in household soil (7.08%) and animal faecal samples (10.67%). This study further revealed that parasitic infections are prevalent among humans, animals, and surrounding soil within shared environments. Our findings highlight the need for improved sanitation, safe water, hygiene education, and sustained disease control.

## 1.0 Introduction

Schistosomiasis and soil-transmitted helminths infections remain among the most prevalent neglected tropical diseases (NTDs) and disproportionately affect marginalized populations in low- and middle-income countries (LMICs), particularly in settings with limited access to adequate water, sanitation, and hygiene (WASH) infrastructure [1]. Globally, schistosomiasis affects an estimated 251 million people, with over 90% of infections occurring in sub-Saharan Africa [2,3]. Schistosomiasis is widely recognized as the second most socioeconomically devastating parasitic infection after malaria in endemic regions [4]. In Ghana, schistosomiasis is primarily attributed to *Schistosoma haematobium* and *Schistosoma mansoni*, which cause urinary and intestinal forms of the disease, respectively, and both significantly contribute to morbidity, particularly among children [5].

Soil-transmitted helminths consist of a group of intestinal parasites transmitted through contact with soil contaminated by infective eggs or larvae, typically in areas with inadequate sanitation and poor waste management [6]. The major species infecting humans include *Ascaris lumbricoides*, *Trichuris trichiura*, *Strongyloides stercoralis*, and the hookworms (*Necator americanus* and *Ancylostoma duodenale*) [7, 8]. Globally, more than 1.5 billion people, representing about 24% of the world’s population, are infected with one or more STHs species, with the highest burden occurring in sub-Saharan Africa, Southeast Asia, and Latin America [7,9]. Children are particularly affected by both infections, commonly suffering from malnutrition, anemia, impaired cognitive development, and reduced school attendance and performance [10,11].

Over the past decade, Ghana has implemented preventive chemotherapy (PC) through mass drug administration (MDA) using praziquantel for schistosomiasis and albendazole or mebendazole for STHs [12]. While these interventions have contributed to reductions in infection intensity and morbidity, transmission persists in many endemic communities, with STHs prevalence ranging from approximately 10% to nearly 50%, and schistosomiasis prevalence varying from low levels in peri-urban areas to over 60% in communities located near rivers, irrigation schemes, and lakeshores [12–15]

Additionally, environmental contamination plays a major role in sustaining helminth transmission [16]. Soil contaminated with helminth eggs and larvae serves as an important route of exposure, particularly for children who often play barefoot or participate in outdoor activities around homes, schools, and communal areas. Furthermore, locations such as latrine surroundings, household compounds, playgrounds, and water collection points may act as localized environmental reservoirs that sustain ongoing transmission cycles [17,18]. Studies across different African settings have reported soil contamination rates ranging from 17.6% to 39.2%, with multiple helminth genera detected, highlighting the risk of ongoing reinfection even in communities that receive periodic mass drug administration [19–21]. However, MDA programmes in Ghana is mainly done at the school-level [22]. Consequently, evidence linking household-level environmental contamination with infection status among children remains limited in many endemic communities.

The Banda District of Ghana is characterized by extensive reliance on rivers and seasonal surface water sources, subsistence agriculture, and inconsistent sanitation coverage, conditions that are favorable for the continued transmission of both schistosomiasis and STHs [23]. Despite over 18 years of annual deworming campaigns in Ghana, available evidence suggests that helminth infections remain endemic in several communities within the district [14,15]. Given the focal nature of helminth transmission, up-to-date prevalence data and identification of household and environmental determinants are essential to inform risk stratification, optimize treatment frequency, and guide integrated control strategies that combine chemotherapy with environmental and behavioral interventions.

This study therefore aimed to determine the prevalence of schistosomiasis and soil-transmitted helminth infections among children in endemic rural communities in the Banda District of Ghana, and to assess household-level environmental determinants associated with helminth transmission. We hypothesized that children exposed to poor sanitation, frequent surface water contact, and environmentally contaminated household areas would have increased odds of schistosomiasis and soil-transmitted helminth infections.

## 2.0 Materials and methods

### 2.1 Ethics statement

Ethical approval was obtained from the Committee for Human Research and Ethics, University of Energy and Natural Resources (approval number CHRE/AP361/025). Permission to conduct the study was granted by the district health directorate and community leaders. Written informed consent was obtained from parents or legal guardians of participating children. In situations where written consent could not be obtained, verbal informed consent was obtained and documented by trained field investigators in accordance with the approved ethical protocol. Verbal or written assent were also obtained from children aged 10–15 years. Participation was entirely voluntary, and all procedures were conducted in accordance with the Declaration of Helsinki and relevant ethical guidelines.

Freshly voided animal faecal samples were collected non-invasively from the animals’ immediate household environment (pens and resting areas) without handling, restraint, or disturbance. As no invasive procedures, physical contact, or experimental manipulation of animals were involved, specific animal ethical approval was not required.

### 2.2 Study area

**Fig 1:**
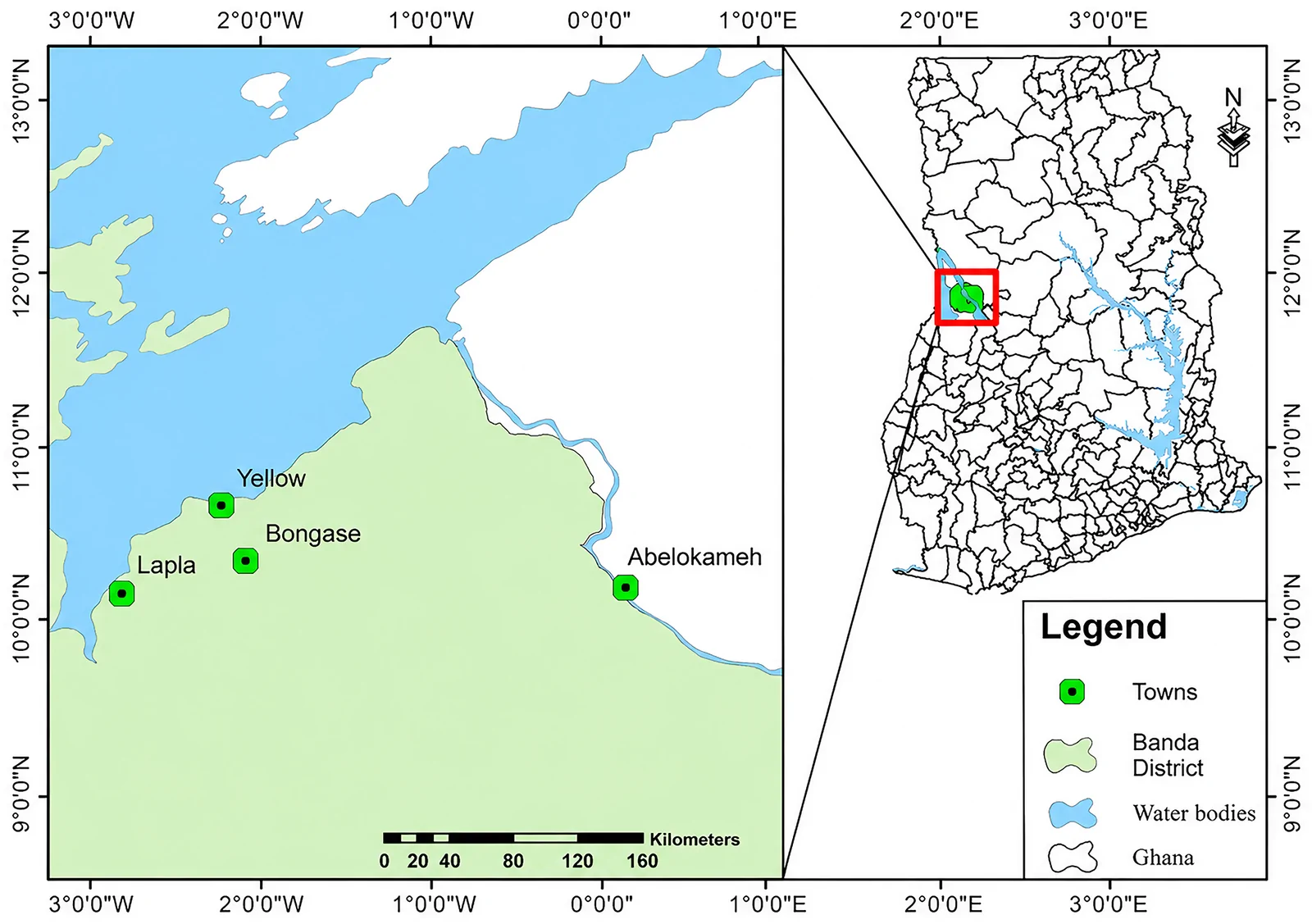
Map of the study area showing the four study communities in the Banda District, Bono Region, Ghana. The inset map shows the location of the Banda District within Ghana. The map was created using ArcGIS Desktop version 10.8.1 (Esri, Redlands, CA, USA) with administrative boundary shapefiles obtained from the Humanitarian Data Exchange (HDX), provided by the United Nations Office for the Coordination of Humanitarian Affairs (OCHA) and the Ghana Statistical Service (March 2021) (https://data.humdata.org/dataset/cod-ab-gha).

This study was conducted in the Banda district in the Bono Region of Ghana, targeting four endemic communities: Banda, Yollow, Agbelokameh, and Lapla, as shown in Fig 1. The District covers an area of approximately 2,298.3 km^2^, and is located between latitudes 7° and 8°45′ North and longitudes 2°52′ and 0°28′ West. As of 2021, it had a population of 28,179, with 41.1% of residents under the age of 15 [23]. The district is entirely rural, where subsistence farming, fishing, and petty trading are the main economic activities. Sanitation infrastructure remains inadequate, as 80.5% of households depend on shared public toilet facilities, 12.8% engage in open defecation, and only 5.4% have access to improved water sources [23]. Among the four selected communities, only Bongase had a health clinic (Bongase Clinic), which is visited occasionally by residents of the other three communities. Routine laboratory investigations, including stool wet-mount microscopy and urine sediment analysis, are undertaken at the clinic. In addition, **annual school-based mass deworming progromme implemented as part of** WHO**-recommended preventive chemotherapy based on infection prevalence thresholds** was conducted under Ghana’s soil-transmitted helminth and schistosomiasis control programme, using albendazole (400 mg) and praziquantel (600 mg) for children aged 7–16 years. [22]. The most recent MDA in the study area occurred in November 2024, prior to our February visit. These environmental, demographic, and sanitary conditions make the Banda District a suitable site for helminth infection study.

### 2.3 Study design and study population

The study employed a community-based cross-sectional design and was conducted between February to April 2025. Children aged 2–15 years, including both school-attending and non-school-attending participants, were selected and screened for schistosomiasis and soil-transmitted helminth infections. Environmental and animal components were also included. soil samples were collected from household surroundings, including, latrine areas, playgrounds and household entry points, while faecal samples from domestic animals were examined soil-transmitted helminths and assess their potential role as reservoirs.

### 2.4 Inclusion and exclusion criteria

Children aged 2–15 years residing in the study communities for at least three months were eligible for inclusion. Written informed consent from parents or guardians and, for children aged 10–15 years, verbal or written assent were required. Children without consent or assent were also excluded.

### 2.5 Sample size estimation

The minimum sample size for the study was estimated using a 95% confidence interval (Z = 1.96), 0.05 margin of error, and an assumed prevalence of 15.45% [5] based on a previous study in similar endemic settings. To account for potential non-responses and data entry errors, a 10% contingency was added, resulting in final a sample size of 410 participants.

### 2.6 Study procedures

Participants were selected using a systematic household sampling approach adapted from established multi-site soil-transmitted helminth survey protocols [24]. Due to the absence of household registers for eligible children and the small size of some communities, participant recruitment across sites was guided by field feasibility without formal proportional allocation. Prior to sampling, community members were informed about the study objectives and planned field activities through traditional gongong beating and announcements at local information centers. In each community, field teams began at a central point, typically the chief’s house. The first household was selected using a simple random method, after which households were visited sequentially through a door-to-door walk and following a systematic route, without prior listing of houses. Within each household, one eligible child aged 2–15 years was randomly selected. If no eligible or consenting child was present; the next household was approached.

### 2.7 Risk factors and demographic data collection

Data on demographic characteristics, including age, sex, school attendance, and risk factors related to water contact activities, sanitation, and hygiene practices associated with helminthiasis, were collected using a pretested structured questionnaire (S1 File) developed in the KoboCollect Toolbox. The questionnaire was adapted from standard instruments used in similar epidemiological studies on soil-transmitted helminths and schistosomiasis. It was piloted in a community with comparable socio-demographic characteristics to the study population, and minor revisions were made based on feedback to improve clarity and consistency. The final version was deployed and administered by trained field assistants. Children aged 10–15 years completed the questionnaire independently or with parental/guardian assistance, while parents or guardians provided responses for children aged 2–9 years.

### 2.8 Biological sampling

Children from the selected endemic communities who provided informed consent were enrolled in the study. Using a global positioning system (GPS) to identify sampling locations, 20–30 mL of urine and 50 g of stool were collected from each participant between 8:00 a.m. and 12:00 p.m. into sterile, labeled containers bearing unique participant codes, date, and time of collection. The samples were immediately placed in a cool box with ice packs and transported from the communities to the laboratory within 5–20 minutes after collection, depending on the distance of the study areas from the Bongase laboratory. All samples were processed within one hour of collection to minimize degradation of helminth ova. Participants with missing laboratory specimens were revisited the following day to complete specimen collection, ensuring complete questionnaire and laboratory data for all enrolled participants.

### 2.9 Environmental sampling

Freshly voided faecal samples (5–10 g) were collected non-invasively from domestic animals (dogs, sheep, goats and chicks) present in each household using sterile spatulas and transferred into sterile, labeled zip-lock bags. Samples were transported from the communities to the laboratory within 5–20 minutes of collection, depending on the distance to the laboratory, and were processed within one hour of collection to minimize degradation of helminth ova. For soil sample collection, in each household, three soil samples were obtained from the area behind the toilet, the household entrance, and the playground area. The top 2–5 cm of soil was collected using sterile spatulas, with approximately 50–100 g obtained per sampling point. Each sample was placed in a clean, labeled container indicating the household identification, sampling location, date, and time of collection. Laboratory analysis was performed within 8 hours of collection.

### 2.10 Laboratory analysis

#### 2.10.1 Urine analysis.

Urine sedimentation method was used to detect *S. haematobium ova*. Briefly, 10 mL of urine samples was centrifuged at 1,500 rpm for 5 minutes, and the sediment was placed on a glass slide, and a drop of Lugol’s iodine was added. Slides were examined using light microscopy at ×10 and ×40 magnifications to detect *the* ova, identified by their terminal spine. Ova were counted per 10 mL of urine, and infection intensity was classified as light (≤50 ova) or heavy (≥50 ova) following WHO guidelines [25].

#### 2.10.2 Stool analysis using the Kato‑Katz examination.

Stool samples were prepared using the Kato-Katz (Sterlitech Corporation, Auburn, WA, USA; Cat. No. 2050020) method following the manufacturer’s instructions. Briefly, each stool sample was homogenized and sieved through a nylon screen onto a glass slide using a pre-labeled template. A glycerol-malachite green cellophane strip was placed over the deposit, and the slide was inverted and pressed to produce a uniform smear. Slides were examined under ×10 and ×40 objectives, and egg counts were extrapolated to eggs per gram (EPG) using multiplication factors based on template mass (×24 for 41.7 mg template). Parasites were identified using the World Health Organization Bench Aid for the Diagnosing of Intestinal Parasites [26]. Infection intensity was classified according to WHO criteria as follows: *A. lumbricoides*, light (1–4,999 EPG), moderate (5,000–49,999 EPG), and heavy (≥50,000 EPG); *T. trichiura*, light (1–999 EPG), moderate (1,000–9,999 EPG), and heavy (≥10,000 EPG); and hookworm, light (1–1,999 EPG), moderate (2,000–3,999 EPG), and heavy (≥4,000 EPG) for each parasite species [27].

#### 2.10.3 Formalin-ethyl acetate concentration technique of animal faeces.

The formalin-ethyl acetate concentration technique described in the WHO Bench Aid for the Diagnosing of Intestinal Parasites [26] was used. Briefly, 1 g of feces was mixed with 10 mL of 10% formalin, fixed for 30 minutes, strained through gauze into centrifuge tubes, and centrifuged at 500 × g for 10 minutes. After discarding supernatant, 7 mL of saline and 3 ml of ethyl acetate were added, shaken, and centrifuged at 500 × g for 3 min. Debris and supernatant were removed, and concentrated sediment was examined microscopically with Lugol’s iodine for ova and larvae.

#### 2.10.4 Flotation examination of soil samples.

To assess environmental contamination with helminth ova, a flotation method adapted from the WHO Bench Aid for the Diagnosing of Intestinal Parasites was used [26]. Briefly, samples were air-dried for 24 hours, sieved through a 250 µm mesh, weighed (1–2 g), mixed with 10% formalin, and allowed to stand for 30 minutes. Suspensions were sieved, centrifuged at 1,500 × g for 5 min, and suspended. Zinc sulfate flotation solution was added to form a meniscus, and a coverslip was placed over it. Coverslips were transferred to glass slides, stained with Lugol’s iodine, and examined under ×10 and ×40 objectives to identify helminth eggs and larvae.

### 2.11 Quality control

Both laboratory and field teams underwent extensive training prior to data collection. Field assistants were trained on questionnaire administration, interviewing techniques, and study procedures to minimize information bias and ensure consistency in data collection. Laboratory technicians were trained using 15 slides containing representative helminth eggs and larvae. Approximately 10% of slides were randomly re-examined daily to verify consistency in parasite identification and egg quantification. All quality control procedures were strictly adhered to at every stage to ensure validity, reliability, and reproducibility of results.

### 2.12 Data analysis

Data were entered into Microsoft Excel 2021 and analyzed using IBM SPSS Statistics version 27.0 (IBM Corp., Armonk, NY, USA). Descriptive statistics were computed to determine prevalence and infection intensity. Infection intensity for *S. mansoni*, *A. lumbricoides*, hookworm, *T. trichiura*, and *Strongyloides* spp. was categorized as light, moderate, or heavy, and for *S. haematobium* as light or heavy, following World Health Organization guidelines. Variables for the multivariable logistic regression model were selected using a forward elimination approach with a liberal p-value threshold of 0.25 at the bivariate analysis stage. Variables meeting this criterion were included in the multivariable model to identify independent predictors.

In multivariate analysis, variables with p ≤ 0.05 were considered statistically significant. Associations were expressed as odds ratios (ORs) and adjusted odds ratios (AORs) with 95% confidence intervals (95% CI). No formal adjustment for multiple comparisons was performed, as the analyses were exploratory and intended to identify potential epidemiological determinants of infection.

## 3.0 Results

### 3.1 Socio-demographic characteristics of participants

Out of the 410 children recruited for the study, the majority (63.4%) were aged 2–9 years, while 36.6% were between 10–15 years. The sex distribution was nearly balanced, with 51% males and 49% females. In terms of schooling, 76.3% of participants were attending school, whereas 23.7% had no formal education. Most participants were Christians (69.5%). The predominant parental occupations were fishing (44.6%) and farming (37.8%). Participants were recruited from four communities, with the highest proportion residing in Bongase (30.2%) and the lowest in Yollow (16.1%), as presented in Table 1.

**Table 1:** Socio-demographic characteristics of participants.

Variable	Frequency (n)	Percentage (%)
**Age**		
2–9	260	63.4
10–15	150	36.6
**Gender**		
Male	209	51.0
Female	201	49.0
**School attendance**		
Formal school	313	76.3
No school	97	23.7
**Religious affiliation**		
Christian	285	69.5
Muslim	122	29.8
Traditionalist	3	0.7
**Parental occupation**		
Fishing	183	44.6
Farmer	155	37.8
Teacher	3	0.7
Trader	65	15.9
None	4	1.0
**Community**		
Bongase	124	30.2
Lapla	111	27.1
Yollow	66	16.1
Agbelokameh	109	26.6

### 3.2 Prevalence of schistosomiasis and soil-transmitted infections among participants by demographics

The prevalence of schistosomiasisand STH infectionsamong the children was analyzed according to age, gender, and school attendance, as presented in Table 2. *A. lumbricoides* infection was significantly higher among children aged 2–9 years compared with those aged 10–15 years (15.4% vs. 8.0%; *p* = 0.030).) *S. haematobium* was slightly higher in older children than in younger children (27.3% vs. 23.4%). Gender-specific analysis revealed that males had a higher prevalence of *S. haematobium* compared with females (28.2% vs. 21.4%), while both sexes exhibited a low prevalence of *S. mansoni* (≤ 2.9%). With regard to school attendance, children not attending school had higher prevalence of *S. haematobium*, *A. lumbricoides*, hookworm, and *T. trichiura* compared with school-attending children (all *p* < 0.05).

**Table 2:** Prevalence of schistosomiasis and soil-transmitted helminthiasis among school-aged children by demographics.

Characteristics	Frequency (n)	*S. haematobium* n (%)	*S. mansoni* n (%)	*A. lumbricoides* n (%)	Hookworm n (%)	*S. stercoralis* n (%)	*T. trichiura* n (%)
**Age group**							
2–9 years	260	61 (23.4)	6 (2.3)	40 (15.4)	19 (7.3)	15 (5.8)	29 (11.2)
10–15 years	150	41 (27.3)	4 (2.7)	12 (8.0)	5 (3.3)	3 (2.0)	9 (6.0)
Total	410	102 (24.9)	10 (2.4)	52 (12.7)	24 (5.9)	18 (4.4)	38 (9.3)
χ² (p-value)	–	0.76 (0.382)	0.05 (0.820)	4.60 (0.030)	2.73 (0.099)	3.22 (0.073)	3.01 (0.083)
**Gender**							
Male	209	59 (28.2)	6 (2.9)	30 (14.4)	14 (6.7)	11 (5.3)	23 (11.0)
Female	201	43 (21.4)	4 (2.0)	22 (10.9)	10 (5.0)	7 (3.5)	15 (7.5)
Total	410	102 (24.9)	10 (2.4)	52 (12.7)	24 (5.9)	18 (4.4)	38 (9.3)
χ² (p-value)	–	2.56 (0.109)	0.33 (0.563)	1.08 (0.300)	0.55 (0.457)	0.77 (0.379)	1.53 (0.216)
**School-attending**							
Non-schooling	97	42 (43.3)	6 (6.2)	32 (33.0)	11 (11.3)	7 (7.2)	20 (20.6)
Schooling	313	60 (19.2)	4 (1.3)	20 (6.4)	13 (4.2)	11 (3.5)	18 (5.8)
Total	410	102 (24.9)	10 (2.4)	52 (12.7)	24 (5.9)	18 (4.4)	38 (9.3)
χ² (p-value)	–	23.07 (< 0.001)	1.52 (0.218)	7.23 (0.007)	13.14 (< 0.001)	14.62 (< 0.001)	13.04 (< 0.001)

χ 2= Chi-square value, p-value < 0.05= Statistically significant.

### 3.3 Species-specific prevalence of helminth infections and Schistosoma–soil-transmitted helminth co-infection among study participants

The overall prevalence of schistosomiasis was 26.3% (108 infections), while the overall prevalence of soil-transmitted helminth infections was 28.0% (115 infections) as presented in Table 3. Among the helminth species identified, *S. haematobium* was the most prevalent, affecting nearly one-quarter of participants (24.9%, 95% CI, 20.77**–**29.36). Among the STH species, *A. lumbricoides* recorded the highest prevalence (12.7%, 95% CI,9.62**–**16.30), with *S. mansoni* being the least prevalent species (2.4%, 95% CI,1.18**–**4.44). A total of 40 participants (9.8%, 95% CI: 7.2**–**13.0) were co-infected with at least one *Schistosoma* species and at least one soil-transmitted helminth.

**Table 3:** Species-specific prevalence of helminth infections and Schistosoma-soil-transmitted helminth co-infection among study participants.

Helminth species	Positive cases(n)	Prevalence (%)	95% CI
*A. lumbricoides*	52	12.7	9.62**–**16.30
*T. trichiura*	38	9.3	6.64**–**12.50
Hookworm	24	5.9	3.79**–**8.58
*S. mansoni*	10	2.4	1.18**–**4.44
*S. Stercoralis*	18	4.4	2.62**–**6.85
*S. haematobium*	102	24.9	20.77**–**29.36
Any Schistosoma-STH co-infection	40	9.8	7.2–13.0

Prevalence calculated using total study population (N = 410). Confidence intervals were estimated using the exact binomial (Clopper-Pearson). Any Schistosoma–STH co-infection was defined as the presence of at least one *Schistosoma* species (*S. haematobium* and/or *S. mansoni*) together with at least one STH species in the same participant.

### 3.4 Intensity distribution of helminth infections among infected children

Among children infected with *S. haematobium* (n = 102), 94.12%, (96) had light infections, whereas 5.88% [6] had heavy infections based on WHO classification. In contrast, all infections due to STHs and *S. mansoni* (n = 145) were of light intensity, with no moderate or heavy infections observed as shown in Table 4.

**Table 4:** Intensity distribution of helminth infections among infected children.

Parasite	Total infected	Light n (%)	Heavy n (%)
*S. haematobium*	102	96 (94.12)	6 (5.88)
STHs + *S. mansoni*	145	145 (100)	0 (0)

Infection intensity was classified according to WHO guidelines [27].

### 3.5 Bivariate and multivariate regression analysis of schistosomiasis across selected factors among children

The bivariate and multivariable logistic regression analyses of schistosomiasis across selected risk factors are presented in Table 5*.* In the bivariate analysis, school attendance, source of drinking water, frequency of surface water contact, gender, and community of residence were associated with schistosomiasis infection and were considered for inclusion in the multivariable model (p ≤ 0.25), while age was not retained. In the multivariable logistic analysis, children who did not attend school had four-fold higher odds of infection compared with those attending school (AOR = 4.59; 95% CI: 2.49**–**8.47; p < 0.001). Similarly, children using rivers or streams as their primary drinking water source had four-fold higher odds of infection than those drinking from pipe-borne water (AOR = 4.58; 95% CI: 1.73**–**12.10; p = 0.009). Community of residence also showed a significant association with infection. Compared with Bongase, children residing in Lapla (AOR = 11.32; 95% CI: 3.40**–**37.67; p < 0.001), Yollow (AOR = 6.36; 95% CI: 1.87**–**21.67; p = 0.003), and Agbelokameh (AOR = 7.06; 95% CI: 2.13**–**23.43; p = 0.001) had higher odds of schistosomiasis infection.

**Table 5:** Bivariate and multivariate analysis of schistosomiasis across selected factors among children.

Categories	Overall (N = 410)	Schistosomiasis		Bivariate Analysis		Multivariate Analysis	
**Schistosomiasis Positive Cases n%**	**Schistosomiasis Negative Cases n%**	**P-value**	**OR (95% CI)**	**P-value**	**AOR (95% CI)**
**Age**				0.417			
10-15	150	43 (28.7)	107 (71.3)		1 (Reference)		**–**
2-9	260	65 (25.0)	195 (75.0)		0.83 (0.53**–**1.30)		**–**
**Gender**				0.120*		0.199	
Male	209	62 (29.7)	147 (70.3)		1 (Reference)		1 (Reference)
Female	201	46 (22.9)	155 (77.1)		0.70 (0.45**–**1.10)		0.72(0.44**–**1.19)
**Does your child go to** **school**				< 0.001*		< 0.001*	
Yes	313	65 (20.8)	248 (79.2)		1 (Reference)		1 (Reference)
No	97	43 (44.3)	54 (55.7)		3.04 (1.87**–**4.93)		4.59 (2.49**–**8.47)
**Source of drinking water**				< 0.001*		0.009*	
Pipe-borne water	52	6 (11.5)	46 (88.5)		1 (Reference)		1 (Reference)
Well	137	21 (15.3)	116 (84.7)	0.507	1.39 (0.53**–**3.66)	0.023	3.66 (1.20**–**11.90)
River/Stream	221	81 (36.7)	140 (63.3)	0.001	4.44 (1.82**–**10.84)	0.002	4.58 (1.73**–**12.10)
**Frequent contact with surface water**				0.002*		0.614	
Never	94	13 (13.8)	81 (86.2)		1 (Reference)		1 (Reference)
Daily	229	77 (33.6)	152 (66.4)	< 0.001	3.16 (1.65**–**6.03)	0.530	1.50 (0.42**–**5.31)
Once a week	51	11 (21.6)	40 (78.4)	0.234	1.71 (0.71**–**4.16)	0.785	1.17 (0.37**–**3.70)
Once a month	36	7 (19.4)	29 (80.6)	0.429	1.50 (0.55**–**4.14)	0.659	0.74 (0.19**–**2.82)
**Community**				< 0.001*		0.001*	
Bongase	124	10(9.3)	114 (37.7)	< 0.001	1 (Reference)		1 (Reference)
Lapla	111	45 (41.7)	66 (29.1)	<0.001	7.77(3.67**–**16.44)	<0.001	11.32 (3.40**–**37.67)
Yollow	66	22 (33.3)	44 (66.7)	<0.001	5.70 (2.50**–**13.00)	0.003	6.36 (1.87**–**21.67)
Agbelokameh	109	31 (28.4)	78 (71.6)	<0.001	4.53 (2.10**–**9.77)	0.001	7.06 (2.13**–**23.43)

OR = odds ratio; AOR = adjusted odds ratio. Variables with p < 0.25 in the bivariate analysis were included in the multivariable logistic regression model. Statistical significance was set at p < 0.05

### 3.6 Bivariate and multivariate regression analysis of soil-transmitted helminth infections across selected factors among participants

The bivariate and multivariable logistic regression analyses of soil-transmitted helminth infections across selected risk factors are presented in Table 6*.* In the bivariate analysis**, age, gender, school attendance, availability of toilet in the household, use of refuse dumping sites in the household and community, handwashing with soap after visiting the toilet, and rearing animals within the compound** were associated with STH infection (p < 0.25) and were therefore included in the multivariable logistic regression model. In the multivariable logistic regression analysis, children aged **2–9 years were two times higher odds of infection compared with those aged 10–15 years** (AOR = 2.18; 95% CI: 1.24**–**3.85; p = 0.007). With respect to gender, **female children had lower odds of infection compared with males** (AOR = 0.56; 95% CI: 0.34**–**0.94; p = 0.027). **Children who did not attend school had nearly six times higher odds of infection compared with those attending school** (AOR = 5.91; 95% CI: 3.41**–**10.24; p < 0.001). Similarly, **children living in households that reared animals within the compound had almost five times higher odds of infection compared with those without animals** (AOR = 4.78; 95% CI: 2.32**–**9.86; p < 0.001).

**Table 6:** Bivariate and multivariatete regression analysis of soil transmitted helminth infections across selected factors among participants.

Categories	Overall (N)	Helminthiasis		Bivariate Analysis		Multivariate Analysis	
**Helminthiasis Positive** **Cases n (%)**	**Helminthiasis Negative Cases n (%)**	**P-value**	**OR (95% CI)**	**P-value**	**AOR (95% CI)**
**Age**				<0.001*		0.007*	
10-15	150	27 (18.0)	123 (82.0)		1 (Reference)		1 (Reference)
2-9	260	88 (33.8)	172 (66.2)		2.33 (1.43**–**3.80)		2.18(1.24**–**3.85)
**Gender**				0.066*			
Male	209	67 (32.1)	142 (67.9)		1 (Reference)	0.027*	1 (Reference)
Female	201	48 (23.9)	153 (76.1)		0.67 (0.43**–**1.03)		0.56(0.34**–**0.94)
**Does your child go to school**				<0.001*		<0.001*	
Yes	313	59 (18.8)	254 (81.2)		1 (Reference)		1 (Reference)
No	97	56 (57.7)	41 (42.3)		5.88 (3.59**–**9.62)		5.91(3.41**–**10.24)
**Availability of toilet in household**				0.004*		0.247	
Yes	141	52 (36.9)	89 (63.1)		1 (Reference)		1 (Reference)
No	269	63 (23.4)	206 (76.6)		0.52(0.34**–**0.82)		0.73(0.43**–**1.25)
**Use of refuse dumping sites in the household and community**				<0.001*		0.001*	
Yes	193	74 (38.3)	119 (61.7)		1 (Reference)		1 (Reference)
No	217	41 (18.9)	176 (81.1)		0.38 (0.24**–**0.59)		0.39(0.23**–**0.66)
**Hand wash with soap after visiting toilet**				0.006*		0.217	
Yes	173	36 (20.8)	137 (79.2)		1 (Reference)		1 (Reference)
No	237	79 (33.3)	158 (66.7)		1.90 (1.21**–**3.00)		1.39(0.82**–**2.36)
**Animals reared within compound**				<0.001*		<0.001*	
No	110	12 (10.9)	98 (89.1)		1 (Reference)		1 (Reference)
Yes	300	103 (34.3)	197 (65.7)		4.27(2.24**–**8.14)		4.78(2.32**–**9.86)
**Infection Outcome**							
**Any of the soil types**				0.569			
Positive	68	21 (30.9)	47 (69.1)		1 (Reference)		
Negative	342	94 (27.5)	248 (72.5)		0.85 (0.48**–**1.50)		–
**Any animal excreta**				0.776			
Positive	32	8 (25.0)	24 (75.0)		1 (Reference)		
Negative	268	61 (22.8)	207 (77.2)		0.88(0.38**–**2.07)		–

OR = odds ratio; AOR = adjusted odds ratio. Variables with p < 0.25 were included in the multivariable model. Animal-faecal analysis used N = 300. Statistical significance was set at p < 0.05.

### 3.7 Prevalence of soil-transmitted helminths in domestic animals

The prevalence of soil-transmitted helminths in domestic animals is presented in Table 7. Overall, 10.67% [32] out of 300 animal samples were positive for any STH ova or larvae. *Strongyloides* spp*.* was the most frequently detected parasite, with the highest in dogs (34.4%; 95% CI: 18.6**–**53.2). *Trichuris* spp*.* was the highest prevalence detected in sheep (4.4%; 95% CI: 1.4-9.9). *Ascaris* spp. was the least prevalent, with higher occurrence in chickens (4.3%; 95% CI: 1.2**–**10.5) than goats (3.2%; 95% CI: 0.4**–**11.3).

**Table 7:** Prevalence of Soil-Transmitted Helminths in Domestic Animals.

Animal (N)	*Strongyloides* spp. n (%), 95% CI	*Trichuris* spp. n (%), 95% CI	*Ascaris* spp. n (%), 95% CI
Dog (32)	11 (34.4%), 18.6**–**53.2	2 (6.3%), 0.8**–**20.8	0 (0.0%), 0.0**–**10.9
Sheep (113)	2 (3.2%), 0.4**–**11.3	5 (4.4%), 1.4**–**9.9	0 (0.0%), 0.0**–**3.2
Goat (63)	3 (2.7%), 0.6**–**7.7	3 (4.8%), 1.0**–**13.4	2 (3.2%), 0.4**–**11.3
Chicks (92)	0 (0.0%), 0.0**–**3.9	0 (0.0%), 0.0**–**3.9	4 (4.3%), 1.2**–**10.5
Total (300)	16 (5.3%), 3.1**–**8.5	10 (3.3%), 1.6**–**6.0	6 (2.0%), 0.7**–**4.3

### 3.8 Distribution of soil-transmitted helminth contamination in household soil

**Fig 2:**
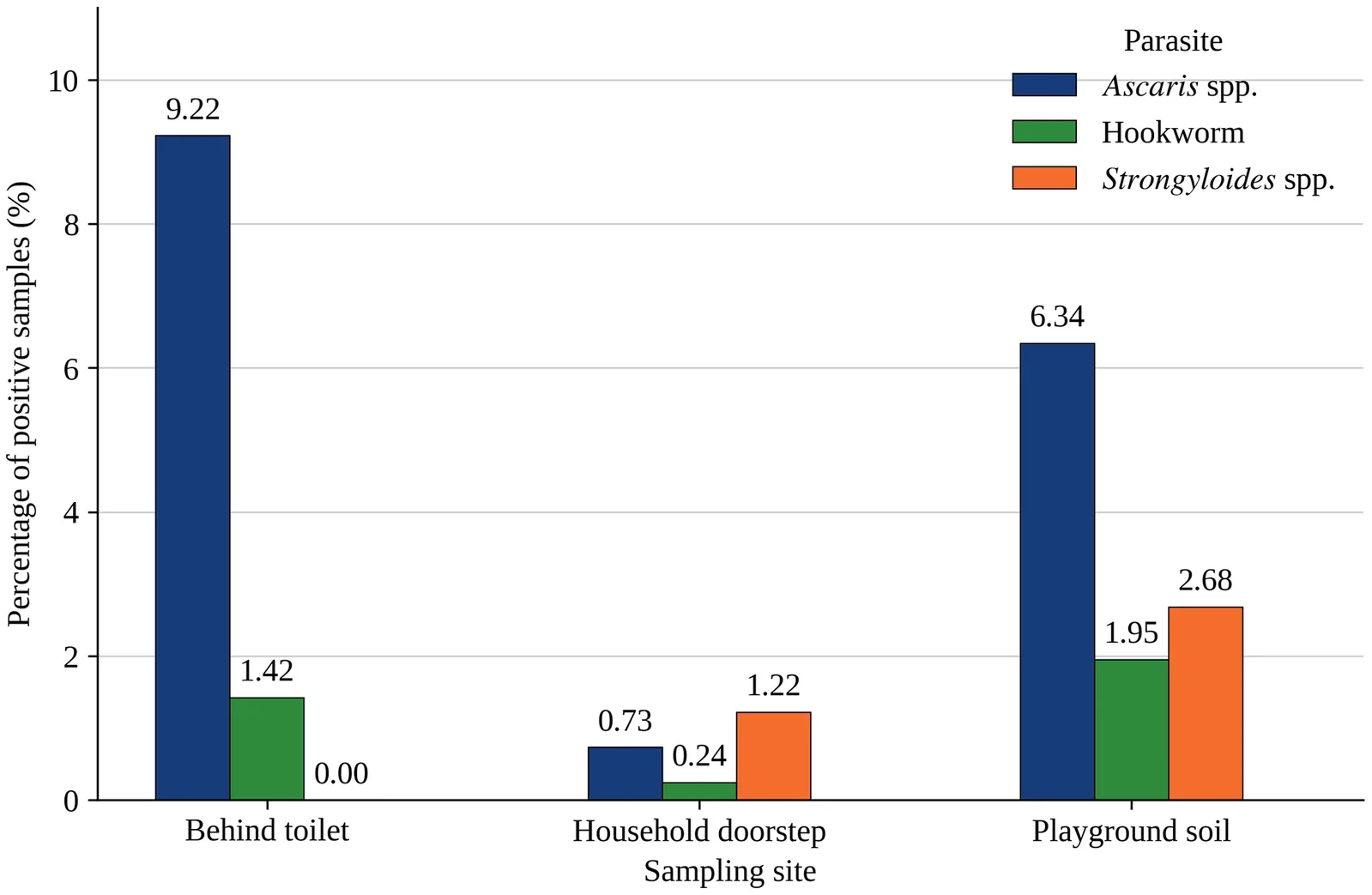
Distribution of soil-transmitted helminths across environmental sampling sites. Grouped bar chart showing the prevalence (%) of *Ascaris* spp., hookworm, and *Strongyloides* spp. in soil samples collected from behind toilets, household doorsteps, and playground soils.

The distribution of soil-transmitted helminth species detected in household soil samples is presented in Fig 2. Overall, 7.08% (68) out of 961 of soil samples were positive for any STHs ova. *Ascaris* spp. 4.37% [42] was the most common species, followed by *Strongyloides* spp.1.66% [16] and hookworms 1.14% [11]. Playground soils (n = 410) contributed the highest absolute number of positive samples, with *Ascaris* spp*.* detected in 6.3% [26] of samples and hookworms in 2.0% [8]. However, soils collected from behind toilet facilities (n = 141) exhibited a higher proportional prevalence of contamination, with *Ascaris* spp. present in 9.22% [13] of samples and hookworms in 1.42% [2]; however, the absolute number of positive samples was lower compared with playground soils. Soil from household doorsteps n = 410 was dominated by *Strongyloides* spp.1.22% [5]

## 4.0 Discussion

Soil-transmitted helminths and schistosomiasis remain among the most prevalent neglected tropical diseases affecting individuals, particularly children in low-resource areas [1]. These infections are driven by inadequate access to safe water, sanitation, and hygiene infrastructure, as well as behavioral factors that increase environmental exposure [28]. In Ghana, national control strategies have focused on **annual mass drug administration** targeting school-aged children [22]. While MDA programs have reduced the burden of infection in several regions, persistent transmission still occurs in certain communities, indicating that chemotherapy alone is insufficient to interrupt transmission [14,29]. The present study aimed to evaluate the current prevalence of schistosomiasis and STHs, identify associated risk factors, and assess household environmental contamination in Banda District to inform targeted interventions.

The overall prevalence of schistosomiasis in this study was 26.3%, indicating a moderate public health burden according to WHO classifications [30]. This prevalence is lower than earlier reports from the Volta Basin in Volta Region of Ghana (46.5%) [31] and Nigeria (40%) [32] but higher than recent national estimates of 4–15% in some regions following sustained MDA [33] in Ghana. Similar prevalence levels have been reported in Côte d’Ivoire (26.1%) [34]. highlighting the focal nature of schistosomiasis transmission. This pattern is likely linked to the close proximity of households to freshwater bodies. These water sources are commonly used by the inhabitants for domestic purposes, fishing, and recreation, leading to frequent contact with cercariae-contaminated water. As a result, children remain vulnerable to reinfection even after treatment.

*S. haematobium* accounted for 24.9% of infections, consistent with its widespread endemicity in Ghana. This aligns with national mapping data in Ghana reporting higher prevalence of *S. haematobium* (20.4%) compared with *S. mansoni* (1.01%) [12]. The observed pattern reflects the distribution of intermediate snail hosts, with *Bulinus* spp. being more widely distributed than *Biomphalaria* spp., thereby sustaining urinary schistosomiasis transmission [35].

The overall STH prevalence was 28%, demonstrating ongoing transmission despite MDA interventions. This is higher than reports from Bosomtwe District in Ghana (19%) [36] and Kenya (24.7%). Among mono-infections, *A. lumbricoides* was the most prevalent species (12.7%), consistent with a recent study in Ghana among pre-school children 12.4% [37] and other African countries, such as in Ethiopia (17.63%) [37] and in Nigeria (39.6%) [38]. The predominance of *A. lumbricoides* is likely due to the resilience of its eggs, which can survive in soil for prolonged periods under favorable environmental conditions [39].

Beyond prevalence, six cases (5.88%) of *S. haematobium* infections were classified as heavy intensity, while all detected STH and *S. mansoni* infections were of light intensity. This indicates that, despite ongoing transmission, the majority of infections occur at low intensity levels. Similar patterns have been reported in other endemic areas where preventive chemotherapy has been implemented, resulting in reduced worm burdens within infected populations [40,41]. Continuous exposure to contaminated water and soil can sustain transmission at low-intensity levels without eliminating the infection. Although infections at low intensity are often less clinically apparent, they remain of public health concern, as they have been associated with adverse outcomes such as anemia and impaired child growth and development [42,43]. The presence of light infection therefore reflects ongoing transmission and highlights the need for sustained control efforts beyond reductions in prevalence alone.

Age-group analysis showed that STH infections were more prevalent among younger children (2–9 years), whereas schistosomiasis was more common among older children. These differences reflect distinct transmission pathways. Younger children are more likely to be exposed through contaminated soil due to play behaviors and poor hygiene practices, while older children have increased exposure to infested water through activities such as swimming, bathing, fishing and assisting with domestic chores [5,7,44]. Males exhibited a higher prevalence of *S. haematobium* than females, the difference was not statistically significant. This is consistent with studies from Nigeria [45] and Uganda [46], where gender differences were attributed to behavioral exposure than rather biological susceptibility.

School attendance was significantly associated with infection status, with non-attending children showing higher prevalence of both schistosomiasis and STH infections. This may reflect reduced access to school-based deworming programs, which form a key component of helminth control strategies [22,47]. Because mass drug administration was delivered at schools shortly before data collection, children attending school likely had higher recent treatment coverage than non-attenders, allowing infections to persist within this population. These untreated individuals may serve as reservoirs for ongoing transmission within the communities.

Environmental factors were also strongly associated with infection risk. Children who relied on rivers or streams water as their primary drinking source had four-fold higher odds of schistosomiasis infection compared with those using piped water. This supports the findings that dependence on untreated surface water increases exposure to cercariae and raises the risk of schistosomiasis [9,10].

Community-level differences in infection prevalence further highlight the focal nature of schistosomiasis transmission. Children residing in Lapla, Yollow, and Agbelokameh had higher odds of infection compared with those in Bongase. Such spatial heterogeneity reflects differences in proximity to contaminated water bodies and varying water-use practices. Such heterogeneity highlights the importance of community-specific intervention strategies.

Schistosoma-soil-transmitted helminth co-infection was observed in the study population, similar findings have been reported in other African studies [38,48]. The co-occurrence of these infections is not unexpected, as they share overlapping environmental and socioeconomic risk factors, including poor sanitation, unsafe water sources, and limited hygiene practices. From a public health perspective, these co-infections are concerning as they may exacerbate morbidity through synergistic effects on nutrition, anemia, and immune function, thereby increasing the overall disease burden in affected populations.

The detection of STHs in 10.7% of domestic animals suggests that these animals may serve as potential environmental reservoirs of helminth contamination. This supports the report that STH infections persist in areas where humans and animals share the same environment, facilitating transmission cycles [49]. *Strongyloides* spp. was the most frequently detected parasite, particularly in dogs, consistent with studies showing that dogs commonly harbor *Strongyloides* spp*.* and may contribute to transmission dynamics in endemic areas [50,51]. The presence of *Trichuris* **spp.** in sheep and goats aligns with their role as natural hosts exposed through grazing in contaminated environments [52]. Although the observed prevalence (4.44.8%) was relatively low, it contrasts with higher report of 29.75% in Kathmandu, Nepal [53], possibly due to differences in animal husbandry practices. Detection of *Ascaris* spp. in goats and chickens has also been reported in previous studies [53,54]. These findings highlight the need for One Health studies to evaluate potential zoonotic transmission.

Although helminth ova/larvae were detected in household soil and animal excreta, no significant association was observed between environmental contamination and infection outcomes, likely reflecting the multifocal nature of contamination across multiple exposure environments. This is supported by Tadege et al. (2022), who reported widespread presence of STH eggs in household, school, and market environments in Jimma Town, Ethiopia [55], demonstrating that exposures extend beyond the domestic areas and involve broader community contact points.

We acknowledge some limitations of our study. Participant recruitment employed a systematic household-based approach rather than a fully probability-based sampling strategy because a complete household sampling frame of eligible children was unavailable. In addition, participants were not allocated proportionally across the study communities according to community population size. These factors may have reduced the representativeness of the study population and introduced selection bias. The analyses did not explicitly account for potential clustering of participants within households or communities, which may result in modest underestimation of standard errors. Sampling was restricted to household sites and may not fully reflect broader community-level contamination and transmission pathways. The cross-sectional design and single time-point sampling preclude causal inference and do not account for seasonal or temporal variations in infection and environmental contamination. T*he use of a single-slide Kato–Katz technique may have underestimated the prevalence and intensity of low-intensity soil-transmitted helminth infections, particularly S. stercoralis, for which Kato–Katz has limited diagnostic sensitivity.* Furthermore, reliance on microscopy-based diagnosis without molecular confirmation may have limited species-level parasite identification. Finally, the lack of adjustment for multiple statistical comparisons may have influenced some of the observed associations. Despite these limitations, this study provides updated epidemiological evidence by integrating human, environmental, and animal data, thereby improving understanding of environmental contamination and informing integrated community-based control strategies.

## 5.0 Conclusion

Schistosomiasis and STH infections remain prevalent among children in the study area despite ongoing preventive chemotherapy. The detection of helminth contamination in both soil and domestic animals highlights the complex, multifactorial pathways of infection within shared household environments. In line with Ghana’s NTD Master Plan and the WHO 2030 NTD Roadmap, integrated approaches combining community-wide deworming, WASH interventions, and environmental surveillance are recommended. **The findings reveal that extending mass drug administration to the community level, beyond school-based programmes, may be necessary to reach non-school-attending children and other at-risk populations.** Future research should incorporate molecular diagnostics and longitudinal monitoring to confirm species identity and investigate zoonotic transmission. **Implementation research is also needed to evaluate the feasibility, acceptability, and cost-effectiveness of integrated community-based intervention approaches in endemic areas.**

## Supporting information

S1 File Raw data underlying the findings reported in the manuscript. This file contains the de-identified raw data underlying the findings reported in this manuscript.
(XLSX)
